# Population Surveillance of Dementia Mortality

**DOI:** 10.3390/ijerph8041244

**Published:** 2011-04-20

**Authors:** Richard F. Gillum, Ralston Yorrick, Thomas O. Obisesan

**Affiliations:** Department of Medicine, College of Medicine, Howard University, 2041 Georgia Ave., Washington, DC 20060, USA; E-Mails: yorrickster@gmail.com (R.Y.); tobisesan@howard.edu (T.O.O.)

**Keywords:** dementia, mortality, geography

## Abstract

Geographic and temporal variation in occurrence of dementia within the US has received little attention despite its importance for generation of new etiologic hypotheses and health services research. We examine methodological problems in the use of vital statistics data for assessing variation over time, among states and within states in the US. We analyzed the US multiple cause of death files for 2005–2006 and 1999–2000 US deaths with Alzheimer’s Disease (International Classification of Disease 10th revision code G30) and other dementias (codes F01, F02, R54) coded as underlying or contributing cause of death based on the death certificate. Age-adjusted death rates were computed by year, state or county for persons aged 65 years and over. In 2005–2006 combined, 555,904 total deaths occurred with any dementia type (212,386 for Alzheimer’s disease) coded as underlying or contributing cause. Among the states, age-adjusted rates per 100,000 per year varied by two fold ranging from 458 in New York to 921 in Oregon. Similar geographic patterns were seen for Alzheimer’s disease. However, between 1999–2000 and 2005–2006 the US death rate for all dementia increased only from 559 to 695 (24%) while that for Alzheimer’s disease doubled from 135 to 266. Use of specific (G30, F01) *versus* non-specific diagnoses (F02, R54) varied among states and over time, explaining most of the temporal increase in rate of Alzheimer’s disease. Further research is needed to assess artifacts of diagnosis, certification or coding, utilization of health services, *versus* biological variation as possible causes of temporal and geographic variation to enhance utility of mortality data for dementia monitoring and research.

## 1. Introduction

Already a major burden in affluent countries, few doubt that population aging trends will continue to increase the burden of dementia worldwide [1,2]. By 2002, Alzheimer’s disease had become the sixth leading cause of death for persons 65 years and over in the US [3]. Many publications have detailed the geographic variation in stroke mortality in the US [4–6]. However, geographic variation in occurrence of dementia within the US has received little attention despite its potential importance for generation of new etiologic hypotheses and health services research. A few authors have reported international variation in dementia prevalence rates [7–10]. The hypotheses are that the rate of death with Alzheimer’s Disease or other dementia varies among states, within states and over time in the US.

## 2. Experimental Section

Data were analyzed from the US multiple cause of death files for 2005–2006 and 1999–2000 [11]. Data from each death certificate were coded to yield a single underlying cause of death and up to twenty multiple causes as well as demographic data. These mortality data were examined for all deaths occurring in the fifty states and the District of Columbia. Deaths of foreign residents and of residents who died abroad were not included. Deaths with Alzheimer’s Disease (International Classification of Disease 10th revision [ICD-10] codes G30.0, Alzheimer's disease with early onset; G30.1, Alzheimer’s disease with late onset; G30.8, Other Alzheimer's disease; G30.9, Alzheimer’s disease, unspecified) coded as underlying or a contributing cause of death were enumerated [12]. Also enumerated were vascular dementia (F01.0, Vascular dementia of acute onset; F01.1 Multi-infarct dementia; F01.2, Subcortical vascular dementia; F01.3, Mixed cortical and subcortical vascular dementia; F01.8, Other vascular dementia; F01.9, Vascular dementia, unspecified); Unspecified dementia (F03); and senility (R54). Death counts shown are totals for each two-year period (1999–2000, 2005–2006). Coding was completed by computer software and does not vary among states. Using the 2000 US standard population, age-adjusted death rates per 100,000 per year (average annual rate for each two-year period) were computed by state or county for persons aged 65 years and over; population estimates and standard methods are documented elsewhere [11]. Maps of death rates were created to show approximate quartiles of state or county rates using mapping software created by the Centers for Disease Control and Prevention [11]. Rates were considered unreliable when based on fewer than 20 deaths (hatched areas on maps). White areas labeled “other” represent counties with fewer than 6 deaths or population less than 100,000. “Background” refers to any areas excluded by the user.

## 3. Results and Discussion

**Figure 1.**
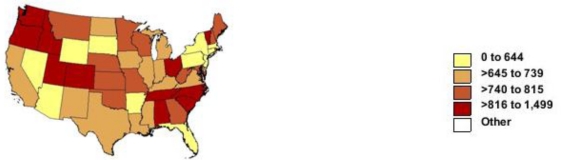
Age-adjusted rate of death with diagnosis of Alzheimer’s disease or other dementias by state for persons aged 65 years and over: United States, 2005–2006.

Results. Among 555,904 with any dementia diagnosis in 2005–2006, 212,386 deaths occurred with Alzheimer’s disease coded as underlying (142,518) or contributing cause (69,868). Among the states, age-adjusted rates per 100,000 for all dementia varied by two fold ranging from 458 in New York to 921 in Oregon (Table 1). Rates varied by over three fold for Alzheimer’s disease ranging from 133 in New York to 419 in Washington (Supplementary Table 1). Figure 1 shows that high rates (>816 per 100,000) for dementia were seen in the Pacific Northwest, some mountain states, Northern New England, East-south central states and the Carolinas. Rates >300 per 100,000 for Alzheimer’s disease were seen in the Pacific Northwest, Northern New England, East-south central states and the Dakotas (Supplementary Figure 1). Low rates were seen in Southern New England and the Mid-Atlantic, Hawaii, Nevada and Florida for both categories.

**Figure 2.**
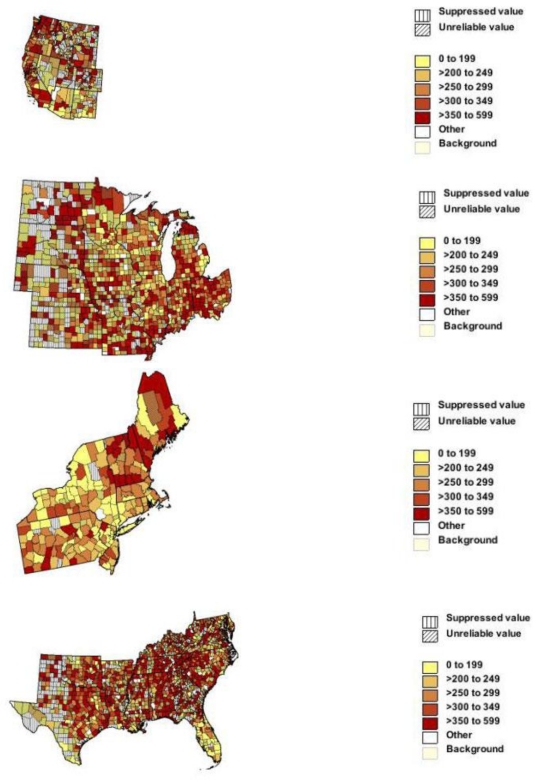
Age-adjusted rate of death with diagnosis of Alzheimer’s disease or other dementia by county for persons aged 65 years and over: United States regions, 2005–2006. Panel A West, panel B Midwest, panel c Northeast, panel D South.

Figure 2 shows death rates for dementia by county. Panel A shows high rates in most Oregon counties, western counties of Washington, and some counties in Mountain states. Alzheimer’s rates were also high in the San Francisco Bay area and much of southern California (Supplementary Figure 2). Panels B and C show high rates in Ohio and northern New England but low rates in most New York counties including New York City and Long Island. Alzheimer’s rates were also low in these areas, but high in the eastern counties of the Dakotas, western Iowa, and Ohio. Panel D shows high rates in many counties in the Appalachian region, Carolinas but low rates in south and central Florida. Alzheimer’s disease showed similar patterns except higher in the Gulf States (Supplementary Figure2). In the US in 2005–2006, death rates per 100,000 for all dementias were higher outside large metropolitan areas (693–759) than in central cities (650) or fringe metropolitan areas (673).

**Figure 3.**
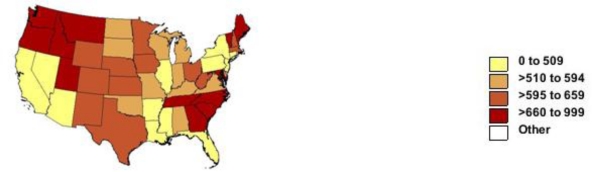
Age-adjusted rate of death with diagnosis of Alzheimer’s disease or other dementias by state for persons aged 65 years and over: United States, 1999–2000.

Between 1999–2000 and 2005–2006, the US death rate per 100,000 for dementia increased from 559 to 695 (24%) but doubled from 135 (92,927 deaths) to 266 for Alzheimer’s disease. Figure 3 shows that geographic patterns were similar to those in 2005–2006 for all dementias. Between 1999–2000 and 2005–2006 for Alzheimer’s disease, Arkansas, Louisiana, Mississippi and Arizona rose markedly in ranking (Supplementary Figure 3); for example, the rate in Louisiana rose from 152 to 320 per 100,000, a larger relative increase but similar absolute increase compared to that in Washington (248 to 419).

To assess the extent to which geographic patterns and trends in Alzheimer’s disease rates might be attributed to variation in diagnostic practices, the numbers of deaths with different diagnostic codes were compared in the US and in states with highest and lowest Alzheimer’s Disease rates (Table 2). Interstate differences were marked with respect to the proportion of deaths assigned to the specific diagnoses of Alzheimer’s disease or vascular dementia *versus* non-specific diagnoses (dementia unspecified, senility); the former were used in 59% of cases in Washington but only 32% of cases in New York. The age-adjusted rate per 100,000 of death for all dementias was 559 in the US, 724 in Washington and 368 New York in 1999–2000 and 695, 840 and 458, respectively, in 2005–2006. Therefore, the difference between Washington and New York in rates of dementia is not merely due to use of difference diagnoses or ICD-10 codes. However, the increase in deaths with any dementia diagnosis in the US or these states between 1999–2000 and 2005–2006 was much less for dementia than that seen for Alzheimer’s Disease, which was mostly due to increasing use of that specific diagnosis over time.

Discussion. This analysis of data from the US multiple cause files is the first to our knowledge to establish inter- and intra-state patterns of geographic variation in death with or Alzheimer’s disease and other dementias. Strengths of this study include its provision of previously lacking data, its use of recent data, its complete coverage of the US population eliminating sampling errors. High rates of death with dementia were seen in the Pacific Northwest, Northern New England, and Southern Appalachia. Low rates were seen in Southern New England and the Mid-Atlantic, Hawaii and Florida. Between 1999–2000 and 2005–2006 use and coding of the diagnosis of Alzheimer’s disease increased so that the US death rate doubled for Alzheimer’s disease but increased only 24% for all dementia.

Mechanisms. Major geographic variation in mortality with dementia could be explained by variations in disease prevalence, a function of incidence and duration. This could result from more sophisticated or accessible care resulting in longer survival with the disease, higher incidence, or both in areas with higher *versus* lower rates. Cardiovascular risk factors such as hypertension have been hypothesized to be risk factors for Alzheimer’s disease and other dementias [12]. However, geographic patterns of stroke mortality were different from those for dementia, with relatively low rates in the Pacific Northwest and northern New England and high rates in the Atlantic Coastal Plain [4]. However, east, and south-central states were high for both. Further, an analysis of trends in stroke mortality predicted that Oregon and Washington, Pacific states, would rise greatly in relative ranking consistent with dementia patterns in 2005–2006 [13]. Like dementia, coronary heart disease mortality was reported to be high in the Ohio River Valley and Carolinas, but, unlike dementia, low in the Pacific Northwest and high in New York [5]. If healthy elderly persons moved from northern New England and Ohio to south and central Florida this could produce higher death rates in the former than in the latter [14,15].

Limitations of this study mean that statistical artifact due to patterns of diagnostic practice and completion of death certificates cannot be excluded as a contributor to geographic and temporal variation in mortality with all dementia [15]. Diagnostic inaccuracy may result in under-ascertainment of specific conditions at death despite introduction of codes for them with ICD-10 [16,17]. Multiple cause data were used to reduce tendency of underlying cause data to underestimate to role of chronic conditions such as dementia on mortality due to immediate causes such as pneumonia and cardiovascular disease. Yet, diagnostic practice may be driven by the availability of specialist care and diagnostic modalities such as magnetic resonance imaging and computerized tomography as well as patients’ access to all these. Lack of personnel and facilities or poor access may tend to increase the use of non-specific diagnoses such as unspecified dementia and senility as well as failure to diagnose dementia before death. Fewer than 10% of deaths from natural causes were autopsied [18]. For deaths occurring outside an institution, the death certificate might be completed by a coroner without physician input. The increasing use of specific diagnoses nationwide seen in this study likely reflects the spread of awareness among physicians and the public and increasing utilization of diagnostic technology among the elderly [2]. Location of dementia research programs might increase awareness in some areas. However, of 30 Alzheimer’s Disease Centers funded by the National Institutes of Health at major medical centers, only 4 were located in the 10 states with the highest Alzheimer’s disease death rates while 7 were located in the 10 states with the lowest rates [19]. California with 6 centers ranked 17th. The current study could not address failure by certifiers to mention chronic dementia at all. When death rates are computed, inaccuracies of population counts and estimates can also limit the utility of data. However, every attempt is made by the U.S. Bureau of Census to understand and compensate for known problems. For example, to accommodate geographic shifts of the Alabama, Louisiana, Mississippi, and Texas populations resulting from Hurricanes Katrina and Rita in 2005, the U.S. Census Bureau developed adjustments in the methodology for state and county population estimates [11]. Aging of the population may not totally be accounted for by age-adjustment, e.g., in trend analysis, since the top bracket was 85 years and over. If elderly persons move from their customary state of residence after dementia diagnosis, their residence at death may not reflect long-term environmental exposure and hence hinder hypothesis generation [20]. Further research addressing these methodological issues would enhance the feasibility of ecological studies of Alzheimer’s disease with respect to known or suspected risk-modifying factors (e.g., hypertension) has been done in the past for cancer [21–23].

## 4. Conclusions

Marked geographic variation in rates of death with all dementia and Alzheimer’s disease occurred among US states and counties and over time. Further research is needed to assess variation in artifacts of certification *versus* variation in disease incidence or duration as possible causes of geographic and temporal variation. Data from multicenter studies of dementia in population-based samples of the US elderly population with validation of death-certificate diagnoses and estimation of incidence, survival and prevalence are needed to establish the magnitude and causes of geographic variation.

## Figures and Tables

**Table 1.** Age-adjusted rates per 100,000 of death with underlying or contributing cause coded as dementia among persons aged 65 years and over: United States, 2005–2006.

**Rank**	**State**	**Count**	**Population**	**Crude Rate**	**Age Adjusted Rate**
1	Oregon	9,721	946,282	1,027	921
2	Ohio	29,421	3,061,308	961	894
3	North Carolina	18,904	2,129,892	888	891
4	Vermont	1,583	164,600	962	886
5	South Carolina	9,376	1,092,416	858	877
6	Utah	3,886	444,628	874	856
7	Washington	13,366	1,457,624	917	840
8	Idaho	2,954	333,258	886	833
9	Alaska	600	90,102	666	832
10	Colorado	7,897	942,768	838	829
11	Alabama	10,097	1,220,292	827	822
12	Tennessee	12,463	1,529,447	815	818
13	West Virginia	4,643	555,956	835	815
14	Minnesota	12,001	1,246,758	963	812
15	Maine	3,346	383,239	873	810
16	Oklahoma	8,016	941,528	851	806
17	Georgia	14,088	1,794,142	785	805
18	Montana	2,246	259,700	865	780
19	Nebraska	4,396	467,522	940	780
20	New Hampshire	2,737	323,149	847	780
21	Wisconsin	12,848	1,448,359	887	772
22	Maryland	10,151	1,291,826	786	767
23	Kansas	6,390	713,211	896	746
24	Rhode Island	2,694	295,308	912	744
25	Missouri	12,626	1,550,721	814	742
26	Iowa	7,985	869,329	919	739
27	Mississippi	5,457	718,511	759	732
28	Texas	34,196	4,608,800	742	732
29	Kentucky	7,736	1,065,217	726	722
30	North Dakota	1,709	185,746	920	714
31	Indiana	11,941	1,560,133	765	712
32	Virginia	12,233	1,751,270	699	702
33	Delaware	1,550	226,190	685	682
34	Michigan	18,202	2,522,361	722	676
35	Louisiana	7,094	1,049,146	676	668
36	California	55,848	7,782,755	718	667
37	New Mexico	3,196	477,957	669	658
38	Illinois	21,985	3,058,693	719	647
39	Wyoming	824	124,420	662	644
40	South Dakota	1,772	222,881	795	637
41	Pennsylvania	27,825	3,768,607	738	635
42	Connecticut	7,120	939,127	758	634
43	Massachusetts	12,690	1,710,356	742	629
44	District Columbia	962	138,952	692	626
45	Hawaii	2,491	354,351	703	621
46	Arkansas	5,014	775,273	647	608
47	Nevada	2,844	545,373	521	604
48	Arizona	9,676	1,551,114	624	602
49	Florida	39,738	6,014,797	661	579
50	New Jersey	13,652	2,252,623	606	547
51	New York	25,714	5,041,320	510	458

**Table 2.** Distribution of deaths with underlying or contributing cause of dementia among persons aged 65 years and over.

		Alzheimer’s Disease		Vascular dementia		Unspec. dementia		Senility		Total dementia	
2005–2006	State	Count	Percent	Count	Percent	Count	Percent	Count	Percent	Count	Percent
	Washington	6,669	48	1,471	11	4,956	36	763	6	13,859	100
	New York	7,405	28	1,004	4	16,120	61	1,951	7	26,480	100
	US	221,386	38	29,251	5	288,237	50	42,879	7	581,753	100
1999–2000	Washington	3,342	41	106	1	4,014	49	751	9	8,213	100
	New York	2,772	18	152	1	10,722	71	1,533	10	15,179	100
	US	92,927	28	2,957	1	193,029	59	40,847	12	329,760	100

## Appendix

Suppl. Figure 1. Age-adjusted rate of death with diagnosis of Alzheimer’s by state for persons aged 65 years and over: United States, 2005–2006.

Suppl. Figure 2. Age-adjusted rate of death with diagnosis of Alzheimer’s disease by county for persons aged 65 years and over: United States regions, 2005–2006. Panel A West, panel B Midwest, C Northeast, panel D South.

Suppl. Figure 3. Age-adjusted rate of death with diagnosis of Alzheimer’s disease by state for persons aged 65 years and over: United States, 1999–2000.

**Suppl. Table 1.** Age-adjusted rates of death with underlying or contributing cause coded as Alzheimer's Disease among persons aged 65 years and over: United States, 2005–2006.

**Rank**	**State**	**Count**	**Population**	**Crude Rate**	**Age Adjusted Rate**
1	Washington	6,669	1,457,624	458	419
2	Tennessee	6,164	1,529,447	403	405
3	North Dakota	890	185,746	479	375
4	Alabama	4,357	1,220,292	357	355
5	South Carolina	3,732	1,092,416	342	349
6	Vermont	598	164,600	363	336
7	South Dakota	904	222,881	406	328
8	Mississippi	2,427	718,511	338	327
9	Oregon	3,384	946,282	358	322
10	Kentucky	3,433	1,065,217	322	321
11	Maine	1,325	383,239	346	321
12	Louisiana	3,384	1,049,146	323	320
13	Ohio	10,513	3,061,308	343	320
14	Idaho	1,115	333,258	335	316
15	North Carolina	6,680	2,129,892	314	315
16	New Hampshire	1,095	323,149	339	312
17	California	25,886	7,782,755	333	309
18	Colorado	2,914	942,768	309	306
19	Arizona	4,889	1,551,114	315	304
20	West Virginia	1,718	555,956	309	302
21	Iowa	3,218	869,329	370	301
22	Oklahoma	2,978	941,528	316	301
23	Missouri	5,041	1,550,721	325	297
24	Indiana	4,960	1,560,133	318	296
25	Texas	13,638	46,088,00	296	293
26	Georgia	5,047	1,794,142	281	289
27	Kansas	2,419	713,211	339	285
28	Arkansas	2,310	775,273	298	281
29	Nebraska	1,530	467,522	327	277
30	Montana	755	259,700	291	264
31	Wisconsin	4,335	1,448,359	299	261
32	Wyoming	334	124,420	268	260
33	Virginia	4,449	1,751,270	254	256
34	Michigan	6,858	2,522,361	272	255
35	District of Columbia	386	138,952	278	250
36	Minnesota	3,587	1,246,758	288	246
37	Rhode Island	886	295,308	300	245
38	Alaska	177	90,102	196	244
39	Illinois	8,250	3,058,693	270	244
40	Pennsylvania	10,086	3,768,607	268	231
41	Delaware	523	226,190	231	230
42	Massachusetts	4,550	1,710,356	266	226
43	Utah	1,008	444,628	227	223
44	Maryland	2,827	1,291,826	219	214
45	Connecticut	2,388	939,127	254	213
46	New Jersey	5,303	2,252,623	235	213
47	New Mexico	985	477,957	206	203
48	Florida	12,597	6,014,797	209	184
49	Nevada	833	545,373	153	176
50	Hawaii	646	354,351	182	161
51	New York	7,405	5,041,320	147	133

**Suppl. Table 2.** Distribution of deaths with underlying or contributing cause of dementia among persons aged 65 years and over: Washington and New York, 1999–2000 and 2005–2006.

		Alzheimer’s Disease		Vascular dementia		Unspec. dementia		Senility		Total dementia	
2005–2006	State	Count	Percent	Count	Percent	Count	Percent	Count	Percent	Count	Percent
	Washington	6,669	48	1,471	11	4,956	36	763	6	13,859	100
	New York	7,405	28	1,004	4	16,120	61	1,951	7	26,480	100
	US	221,386	38	29,251	5	288,237	50	42,879	7	581,753	100
1999–2000	Washington	3,342	41	106	1	4,014	49	751	9	8,213	100
	New York	2,772	18	152	1	10,722	71	1,533	10	15,179	100
	US	92,927	28	2,957	1	193,029	59	40,847	12	329,760	100
