# Supplementary figures and images for: Population Surveillance of Dementia Mortality
Source: Int J Environ Res Public Health. 2011 Apr 20;8(4):1244–57. doi: 10.3390/ijerph8041244 (PMC3118887; doi:10.3390/ijerph8041244)

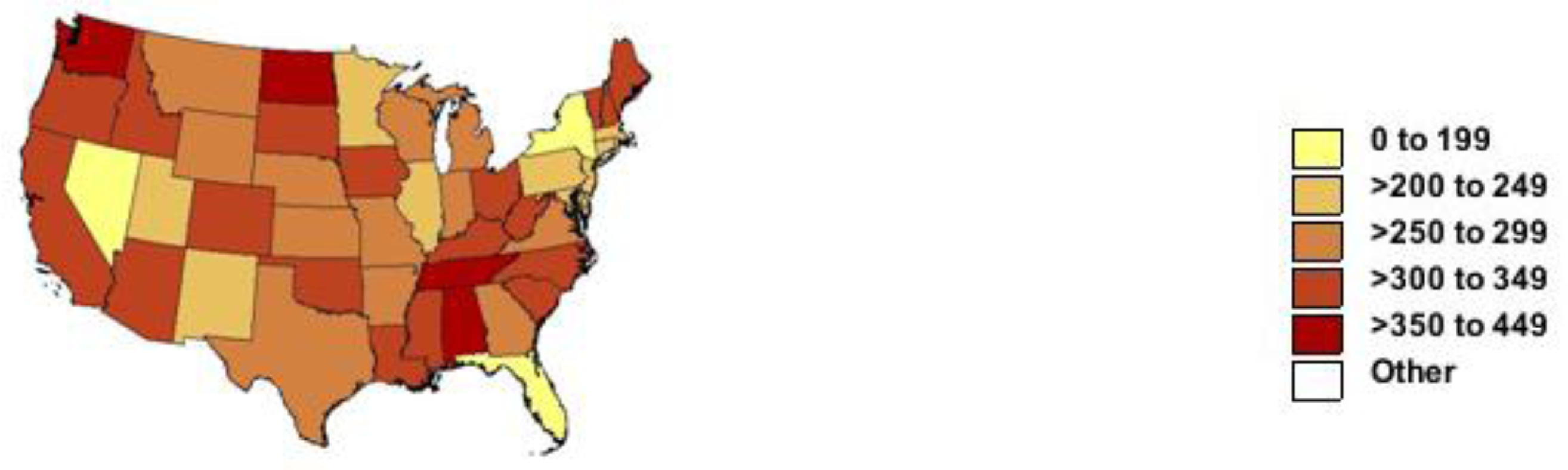

Supplement: Suppl. Figure 1. — Age-adjusted rate of death with diagnosis of Alzheimer’s by state for persons aged 65 years and over: United States, 2005–2006. [file ijerph-08-01244s001.tif]

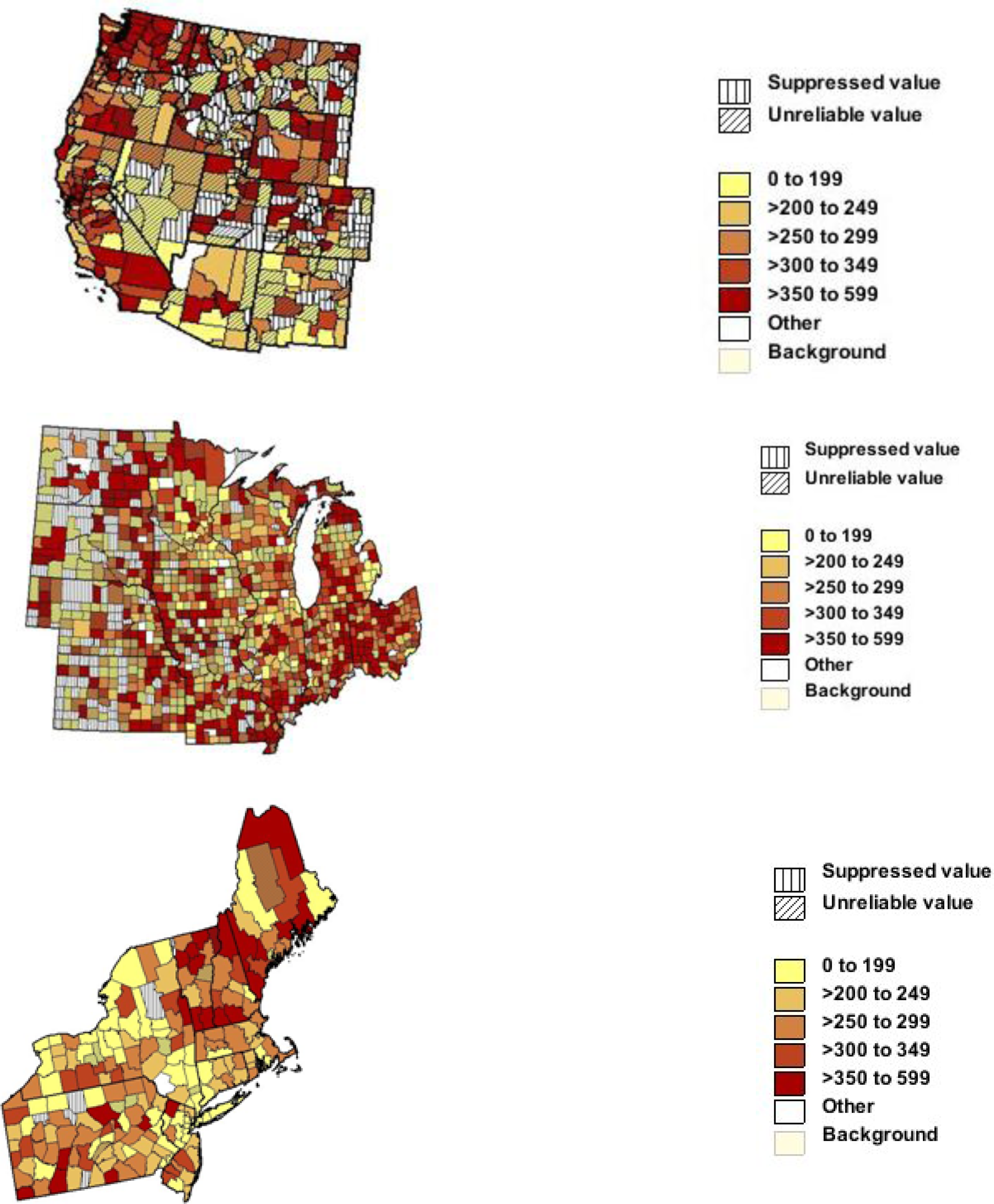

Supplement: Suppl. Figure 2. — Age-adjusted rate of death with diagnosis of Alzheimer’s disease by county for persons aged 65 years and over: United States regions, 2005–2006. Panel A West, panel B Midwest, C Northeast, panel D South. [file ijerph-08-01244s002a.tif]

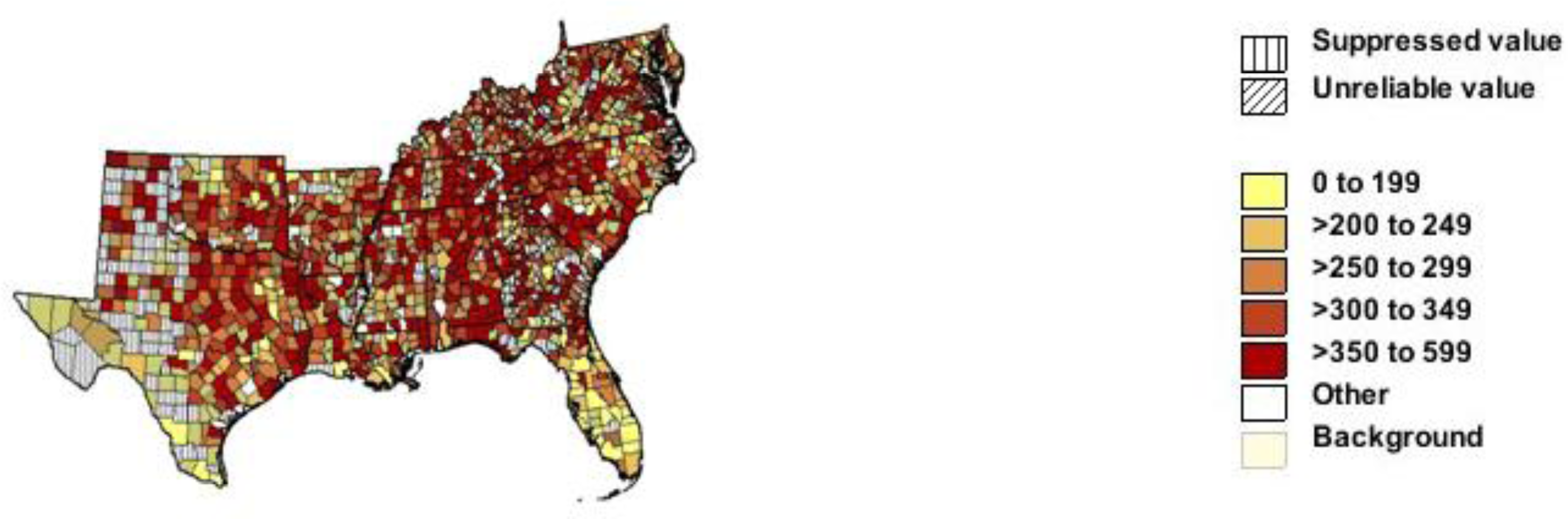

Supplement: Suppl. Figure 2. — Age-adjusted rate of death with diagnosis of Alzheimer’s disease by county for persons aged 65 years and over: United States regions, 2005–2006. Panel A West, panel B Midwest, C Northeast, panel D South. [file ijerph-08-01244s002b.tif]

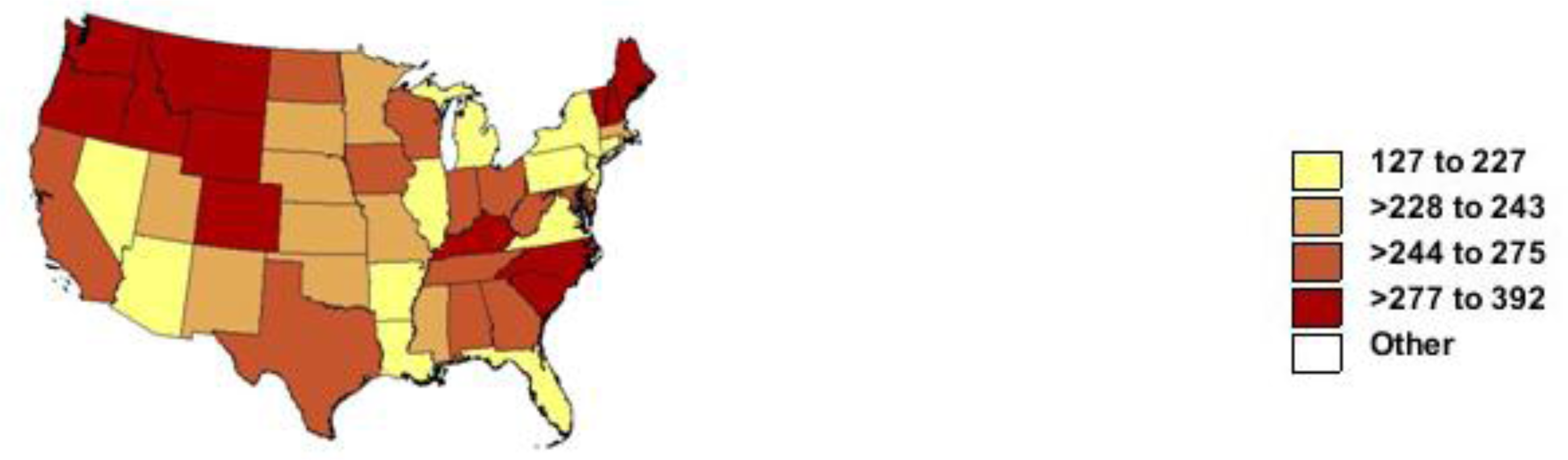

Supplement: Suppl. Figure 3. — Age-adjusted rate of death with diagnosis of Alzheimer’s disease by state for persons aged 65 years and over: United States, 1999–2000. [file ijerph-08-01244s003.tif]
